# Intestinal anastomotic injury alters spatially defined microbiome composition and function

**DOI:** 10.1186/2049-2618-2-35

**Published:** 2014-09-15

**Authors:** Benjamin D Shogan, Daniel P Smith, Scott Christley, Jack A Gilbert, Olga Zaborina, John C Alverdy

**Affiliations:** 1 Department of Surgery, University of Chicago, 5841 S. Maryland, Chicago, IL 60637, USA; 2 Institute for Genomic and Systems Biology, Argonne National Laboratory, 9700 South Cass Avenue, Argonne, IL 60439, USA; 3 Department of Ecology and Evolution, University of Chicago, Chicago, IL 60637, USA; 4 Current address: Center for Metagenomics and Microbiome Research, Baylor College of Medicine, Houston, TX 77030, USA

**Keywords:** Colon anastomosis, 16S rRNA, PiCRUST, Bacterial composition, Predicted function, Anastomotic tissues, Luminal content

## Abstract

**Background:**

When diseased intestine (i.e., from colon cancer, diverticulitis) requires resection, its reconnection (termed anastomosis) can be complicated by non-healing of the newly joined intestine resulting in spillage of intestinal contents into the abdominal cavity (termed anastomotic leakage). While it is suspected that the intestinal microbiota have the capacity to both accelerate and complicate anastomotic healing, the associated genotypes and functions have not been characterized.

**Results:**

Using 16S rRNA amplicon sequencing of samples collected on the day of surgery (postoperative day 0 (POD0)) and the 6th day following surgery (postoperative day 0 (POD6)), we analyzed the changes in luminal versus tissue-associated microbiota at anastomotic sites created in the colon of rats. Results indicated that anastomotic injury induced significant changes in the anastomotic tissue-associated microbiota with minimal differences in the luminal microbiota. The most striking difference was a 500-fold and 200-fold increase in the relative abundance of *Enterococcus* and *Escherichia/Shigella*, respectively. Functional profiling predicted the predominance of bacterial virulence-associated pathways in post-anastomotic tissues, including production of hemolysin, cytolethal toxins, fimbriae, invasins, cytotoxic necrotizing factors, and coccolysin.

**Conclusion:**

Taken together, our results suggest that compositional and functional changes accompany anastomotic tissues and may potentially accelerate or complicate anastomotic healing.

## Background

Removal (resection) of a segment of the gastrointestinal tract is a common procedure performed by general surgeons for a variety of illnesses including cancer, diverticulitis, or intestinal obstruction. When surgeons resect a diseased segment of the gastrointestinal tract, intestinal continuity is reestablished by carefully suturing or stapling the remaining ends together to create a viable and well-sealed connection referred to as an ‘anastomosis’. An anastomosis therefore is a significant tissue injury given that the surgeon must first divide all of the blood vessels feeding the proposed resection site; all layers of the intestinal wall are divided meaning that their entire thickness from mucosa to serosa is disrupted and then tissues are approximated with sutures and staples that represent foreign bodies embedded into the area of healing tissues. As such, surgeons routinely administer antibiotics directed against the intestinal microbiota to decontaminate the intestine of its microflora in an effort to reduce the presumed effect of the microbiota on healing [1]. Despite excellent suture technique by highly skilled surgeons to approximate the tissues and fuse and seal the intestinal wall, without proper subsequent healing of the anastomotic tissues, ‘leak’ can occur resulting in spillage of intraluminal contents into the peritoneal cavity that is associated with sepsis, permanent need for an ostomy, and death. Thus, despite excellent surgical technique, leaks still occur and its pathogenesis remains unknown. While surgeons have known for over 60 years that the intestinal microbiota play a key and critical role in the etiopathogensis of leakage [2], the species involved and the molecular details of their activity remain unknown. Work from our lab has recently confirmed that both the species and phenotype of microbes present on anastomotic tissues is a critical determinant of anastomotic leakage following intestinal resection and anastomosis [3]. In this study, we identified that when high swarming phenotypes of *Pseudomonas aeruginosa* are present on anastomotic tissues, they are associated with leaks. Yet, the choice of antibiotics to provide prophylaxis to patients is based on culture-derived results of the luminal microbiota before and after antibiotic administration that are now several decades old. Because anastomotic tissues are generally inaccessible, it is extremely difficult to track changes in microbial content over the course of healing. As a result, it is not known whether the community structure, membership, and/or function of the luminal microbiota are representative of the microbiota that associate with anastomotic tissues themselves. Furthermore, it is now recognized that culture-based studies of the luminal microbiota fail to classify the majority of the microbial diversity present. Finally, genetic sequencing has revealed the dependency of intestinal microbial community structure, membership, and function on the regional and spatial context in which they establish niche specialization [4,5]. Therefore, there is a significant gap in knowledge as to whether healing anastomotic tissues harbor a distinct microbial community from the luminal microbial community. Elucidation of this issue may have a significant impact on the rationale for choosing antibiotics in preparation for colon surgery.

Therefore, the aims of this study were to create anastomotic injury in rats similar to that which is performed in humans and to determine the changes and differences in the luminal versus tissue microbiota over a 6-day course of anastomotic healing.

## Results

### Anastomosis induces changes in the composition of the intestinal microbiota associated with intestinal tissue, but does not affect the microbiota associated with luminal contents

**Figure 1:**
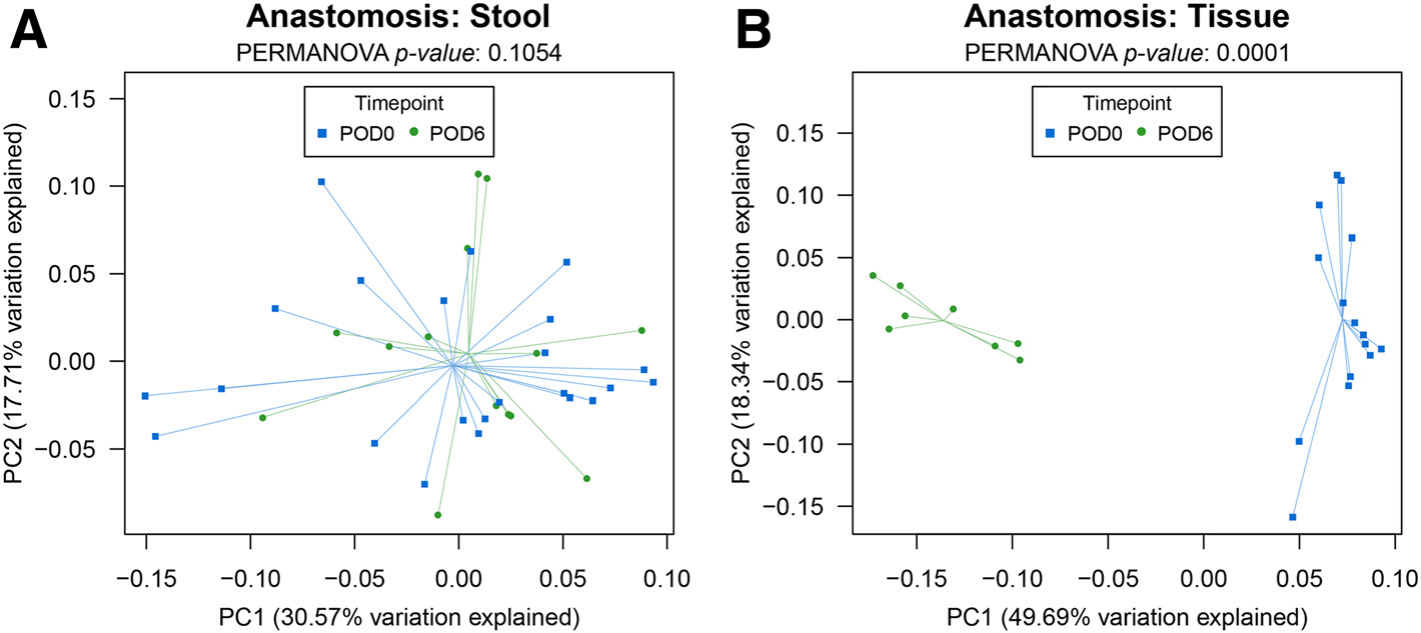
**Principal components analysis of luminal- and tissue-associated microbiomes.** Colon surgery affects the composition of the intestinal microbiota associated with intestinal tissue **(B)** but not the microbiota associated with luminal contents **(A)**. *n* = 10 per group. PERMANOVA *p* values based on 10,000 random permutations of the dataset. Multiple comparisons were performed using the Bonferroni method. *Error bars* = ±2 standard error.

Rats were subjected to removal (resection) of 1 cm of colon just above the colorectal junction followed by reconnection (anastomosis) as is typically performed in humans with colon cancer of the distal intestine. DNA was isolated from resected colon and colon luminal content at the time of surgery, represented as postoperative day 0 (POD0). On postoperative day 6 (POD6), rats were sacrificed and the luminal and tissue associated microbiota at the anastomotic segment were harvested for analysis. Results demonstrated that colon surgery did not affect the microbial community structure associated with the luminal contents (stool), but induced significant changes in the composition of the intestinal microbiota associated with the intestinal tissue (Figure 1A,B).

### The tissue microbiota at POD6 is enriched by commensal opportunistic bacteria

**Figure 2:**
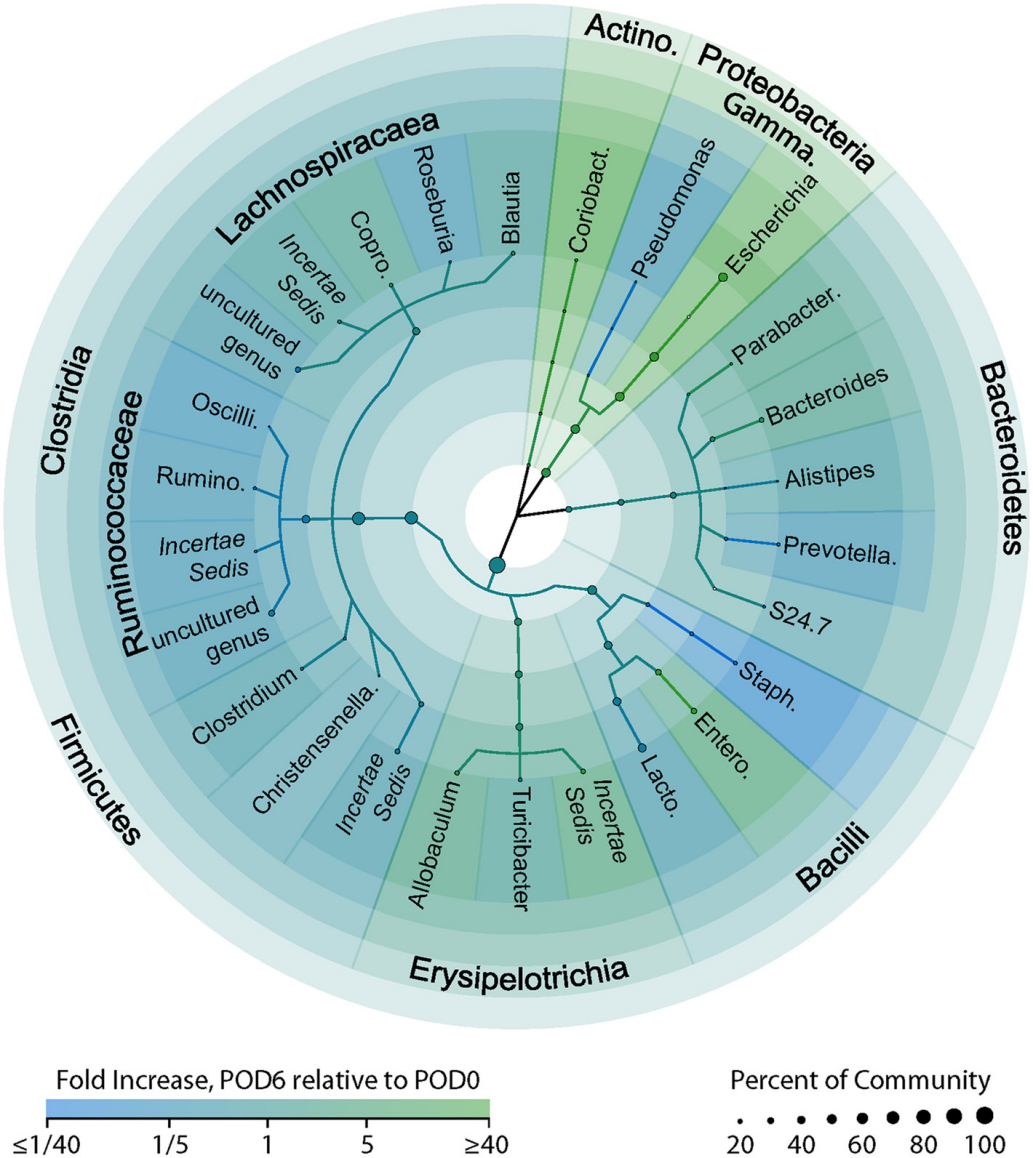
**Taxonomic tree of anastomotic tissue microbiome changes.***Shading* indicates increases (*green*) and decreases (*blue*) in relative abundances of taxonomic groups on POD6 relative to POD0. *Point sizes* represent the higher point-in-time average abundance of the group relative to the entire microbial community. Groups comprising less than 1% of the total community on POD0 and POD6 are excluded.

**Figure 3:**
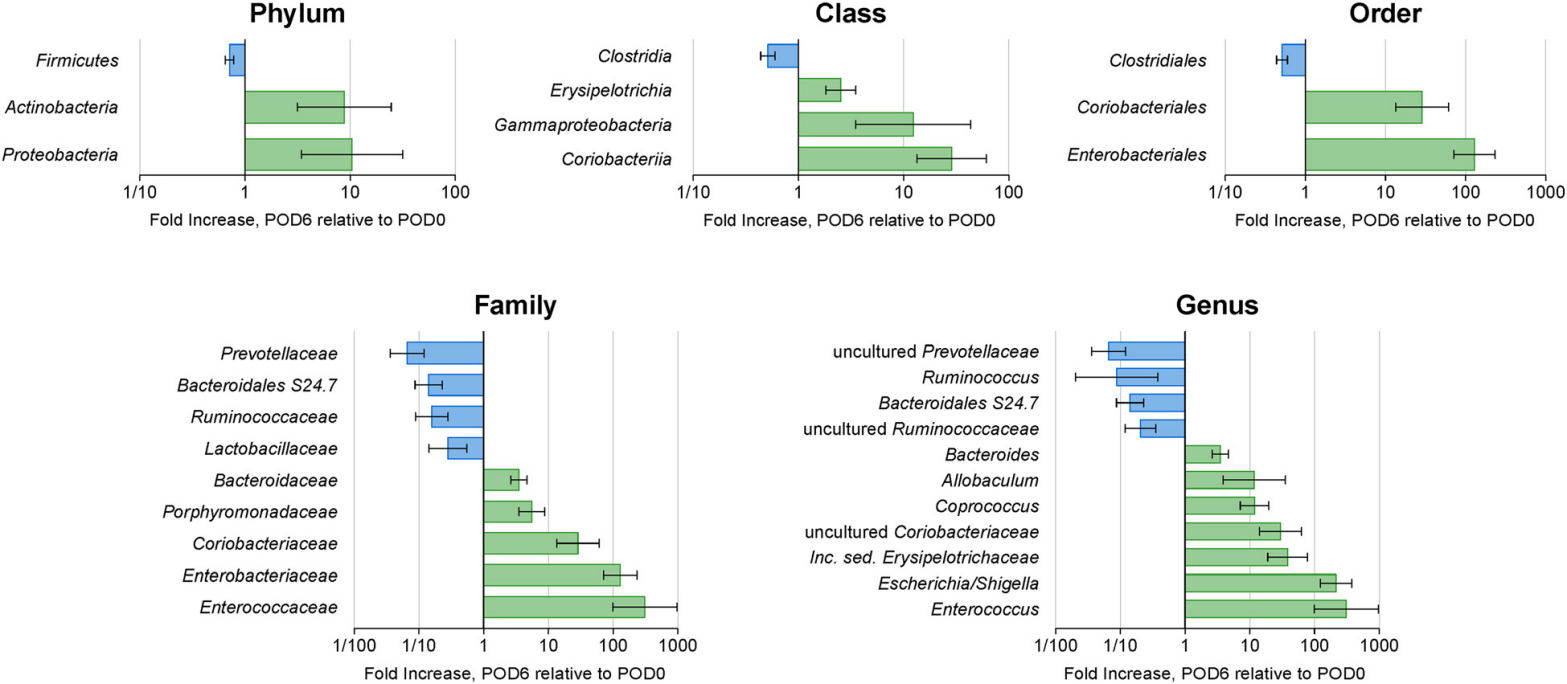
**Comparative analysis of bacterial abundance in anastomotic tissues at POD0 and POD6.** The analysis is presented at phylum, order, class, family, and genus levels.

The taxonomic profiling of tissue-associated microbiota demonstrated that most of the changes in bacterial composition were observed in the phyla Proteobacteria, Actinobacteria, and Firmicutes (Figure 2). Among Proteobacteria, the relative abundance of *Escherichia/Shigella* (c_*Gammaproteobacteria*;o_*Enterobacteriales*;f_*Enterobacteriaceae*) increased 200-fold. In Actinobacteria, the relative abundance of an uncultured bacterium (c_*Coriobacteria*;o_*Coriobacteriales*;f_*Coriobacteriaceae*) increased approximately 50-fold (Figure 3). Non-uniform changes among the genera Firmicutes were observed. The relative abundance of uncultured bacteria representing *Ruminococcaceae* and *Clostridia* decreased 20-fold, while the relative abundance of the genera *Allobaculum* and *Coprococcus* increased approximately 10-fold, and *Enterococcus* increased 500-fold (Figure 3). Finally, a 20-fold decrease in the relative abundance of *Prevotellaceae*, demonstrated that Bacteroidetes were also influenced by anastomotic injury (Figure 3).

### Predicted functional profiling suggests the potential of virulence-associated pathways in post-anastomotic tissues

**Figure 4:**
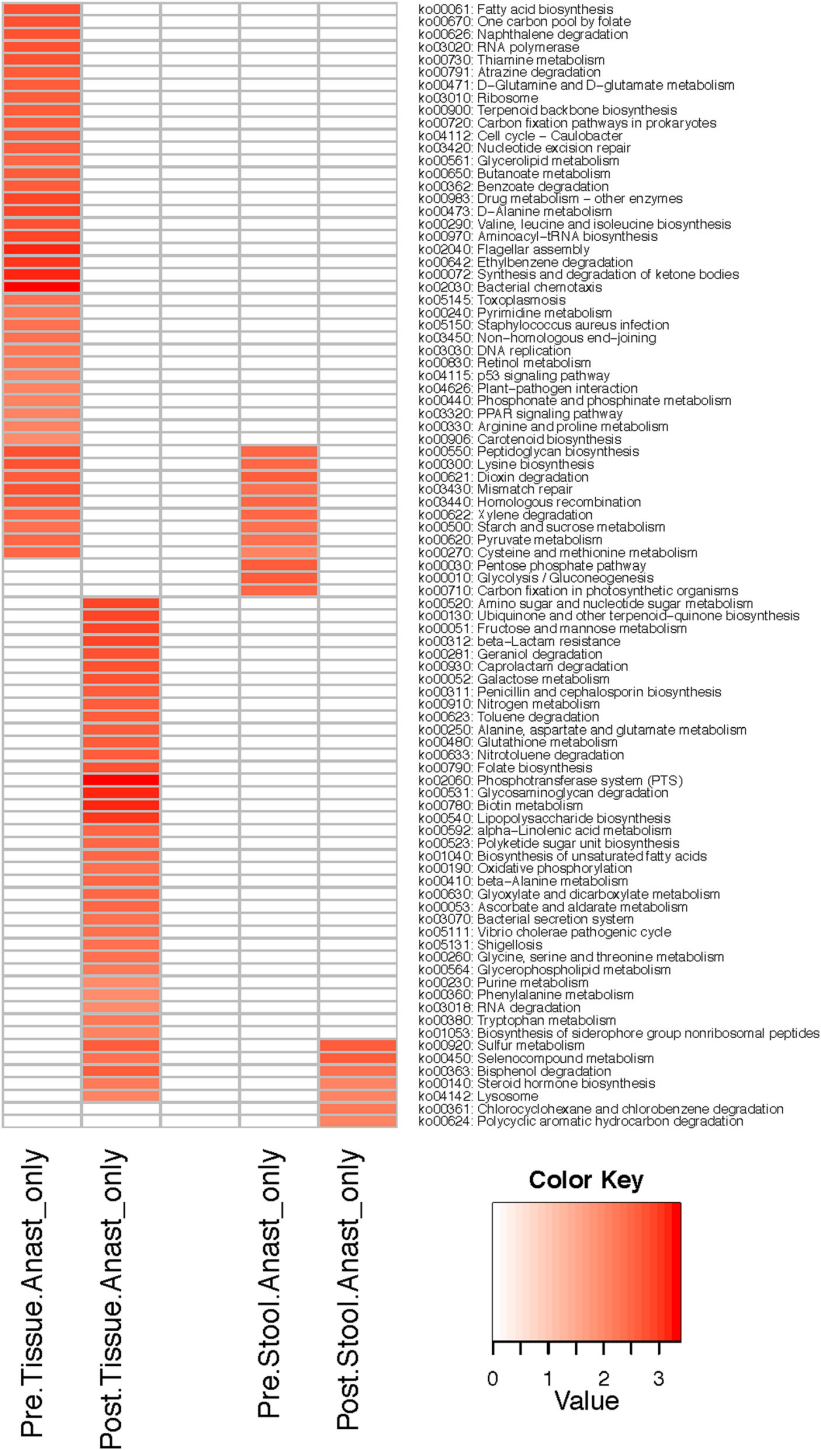
**Enriched metabolic pathways for pre- and post-anastomic tissue and stool.** Pathway enrichment for KEGG metabolic pathways using HUMAnN followed by statistical comparative analysis using LEfSe was performed to determine differential enrichment between POD0 and POD6 for tissue and stool. *Red color* indicates that the KEGG metabolic pathway is enriched in comparison to the corresponding POD for either tissue or stool. Tissue and stool were not directly compared with each other, so common metabolic pathways do not indicate equivalent levels of enrichment for both.

**Figure 5:**
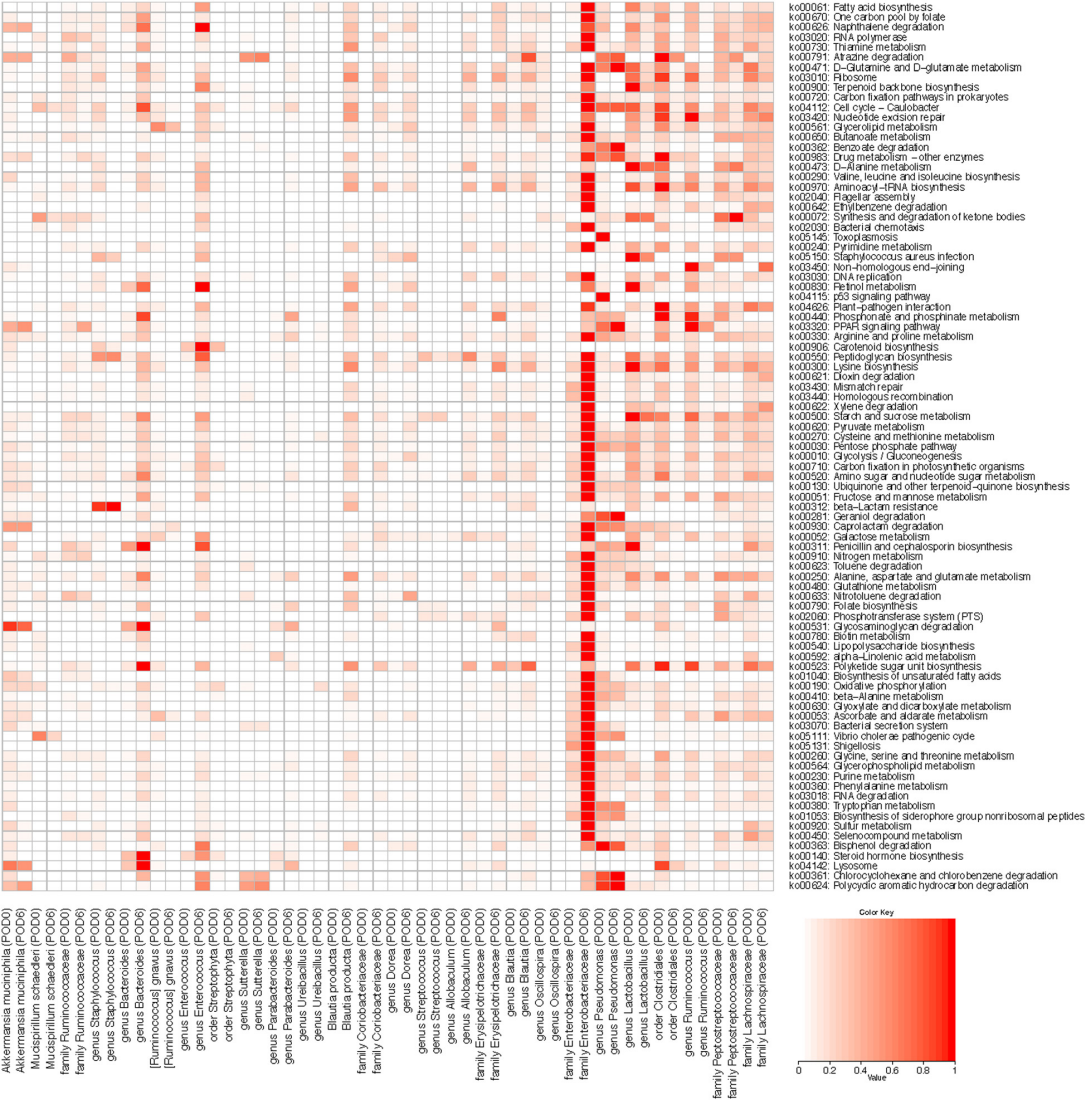
**Contribution of taxonomic groups to the enriched metabolic pathways for pre- and post-anastomotic tissue.** There is a shift in the primary contributors to the metabolic functions from taxa such as genus *Lactobacillus*, order *Clostridiales*, and genus *Ruminococcus* for pre-anastomotic tissue to taxa such as family *Enterobacteriaceae*, genus *Enterococcus*, and genus *Bacteroides* for post-anastomotic tissue. Values are scaled to a 0–1 range for each KEGG metabolic pathway (ko) individually, with 1 indicating the highest contributing OTU and 0 indicating no contribution by the OTU.

The functional potential of the microbiota associated with the intraluminal contents (stool) and tissue before (POD0) and after anastomosis (POD6) was predicted using PiCRUST [6]. There were no statistically significant functional differences for the intraluminal contents between POD0 and POD6, while the microbiota associated with intestinal tissue revealed multiple significantly altered functional attributes after anastomosis (Table 1). The upregulated functions in tissue-associated microbiota can be traits of *Escherichia* and *Enterococcus*. The virulence-associated functions such as pore formation by hemolysin E [7], bacterial attachment to mammalian cells via AIDA-I adhesin-like protein [8], cytotoxicity to eukaryotic cells by cytolethal toxins [9] and cytotoxic necrotizing factor 1 [10], increased motility (outer membrane usher protein, fimbrial-like protein, type 1 fimbrial protein, fimbrial chaperone protein) [11], and invasion of epithelial cells via invasion B [12] can be attributed to *Enterobacteriaceae*, particularly, *Escherichia*. The proteolytic cleavage by coccolysin also known as gelatinase can be attributed to *Enterococcus*[13,14]. Importantly, gelatinase GelE of *Enterococcus faecalis* has been shown to degrade collagen, a critical component for anastomotic tissue healing [15,16]. We further performed enrichment analysis for KEGG metabolic pathways to determine what metabolic changes occur at post-anastomotic tissues versus stool. The analysis indicates there are substantially more metabolic changes for tissue-associated microbiota than for the microbiota in the luminal contents, as expected based on changes in microbial community structure (Figure 4). Furthermore, the analysis of post-anastomotic tissues predicts virulence-associated metabolic pathways such as lipopolysaccharide biosynthesis, bacterial secretion system, and shigellosis, which are not represented in the stool microbiota. The metabolic pathways were linked to the OTUs responsible for the functions (Figure 5). The analysis demonstrated a shift in the primary contributors to the metabolic functions such as genus *Lactobacillus*, order *Clostridiales*, and genus *Ruminococcus* for pre-anastomotic tissue versus family *Enterobacteriaceae*, genus *Enterococcus*, and genus *Bacteroides* for post-anastomotic tissue (Figure 5).

**Table 1:** Predicted upregulated functions in tissue-associated microbiota after anastomosis

**KEGG orthology**	**Description**	**Pathway/function**	**Primary contributor**	***p *** **value**
K03276	UDP-glucose/galactose:(glucosyl)LPS alpha-1,2-glucosyl/galactosyltransferase [EC:2.4.1.-]	Lipopolysaccharide biosynthesis	*Enterobacteriaceae*	0.02
K11139	Hemolysin E	Pore-forming toxin [7]	*Enterobacteriaceae*	0.02
K03765	Transcriptional activator of cad operon	The cad operon encodes a system for neutralization of low extracellular pH	*Enterobacteriaceae*	0.02
K12678	AIDA-I adhesin-like protein	Autotransporter family porin, mediates bacterial attachment to mammalian cells [8]	*Enterobacteriaceae*	0.02
K09925	Hypothetical protein			0.02
K11014	Cytolethal distending toxin subunit B (CdtB)	Initiates a eukaryotic cell cycle block at the G2 stage prior to mitosis, CdtB potentiates a cascade leading to cell cycle block [9]	*Enterobacteriaceae*	0.02
K11015	Cytolethal distending toxin subunit C (CdtC)	CdtC and CdtA function as dimeric subunits, which bind CdtB and delivers it to the mammalian cell interior	*Enterobacteriaceae*	0.02
K11013	Cytolethal distending toxin subunit A (CdtA)	Same as above	*Enterobacteriaceae*	0.02
K11264	Methylmalonyl-CoA decarboxylase [EC:4.1.1.41]	Propanoate metabolism	*Enterobacteriaceae*	0.02
K13244	c-di-GMP-specific phosphodiesterase [EC:3.1.4.52]	Hydrolases acting on ester bonds	*Enterobacteriaceae*	0.02
K03459	Formate transporter (focB)	Electrochemical potential-driven transporters	*Enterobacteriaceae*	0.02
K07354	Outer membrane usher protein (sfmD)	Bacterial motility proteins [11]	*Enterobacteriaceae*	0.02
K07355	Fimbrial-like protein (sfmF)	Bacterial motility proteins	*Enterobacteriaceae*	0.02
K07352	Type 1 fimbrial protein (sfmA)	Bacterial motility proteins	*Enterobacteriaceae*	0.02
K07353	Fimbrial chaperone protein	Bacterial motility proteins	*Enterobacteriaceae*	0.02
K12985	(Galactosyl)LPS 1,2-glucosyltransferase [EC:2.4.1.-]	Lipopolysaccharide biosynthesis proteins	*Enterobacteriaceae*	0.02
K10708	Fructoselysine 6-phosphate deglycase [EC:3.5.-.-]	Hydrolases acting on carbon-nitrogen bonds, other than peptide bonds	*Enterobacteriaceae*	0.02
K11008	Cytotoxic necrotizing factor 1 (cnf1)	Blocks cell cycle G2/M transition in uroepithelial cells [10]	*Enterobacteriaceae*	0.02
K11920	AraC family transcriptional regulator (envY)	DNA-binding transcriptional regulator	*Enterobacteriaceae*	0.02
K13285	Invasin B	Bacterial invasion of epithelial cells [12,17]	*Enterobacteriaceae*	0.03
K08605	Coccolysin [EC:3.4.24.30] (gelE)	Metallopeptidases [13,14]	*Enterococcus*	0.03

## Discussion

Advances in non-culture-based analyses of the intestinal microbiome have demonstrated that environmental influences (e.g., diet, antibiotic therapy, age, colonization history, geography) have profound effects on microbiota community structure, membership, and function [18], which are region specific within the gastrointestinal tract [4,5]. Although intestinal surgery in the form of resection and anastomosis has been practiced for decades, until now, there has been no systematic assessment of the bacterial diversity and functional potential associated with this surgery. It has been observed in germ-free and antibiotic-treated animals that disruption of the normal microbiota results in a weaker intestinal anastomosis as measured by burst strength (i.e., the pressure at which an anastomosis will burst) [19,20]. In these studies, they also observed a loss of collagen, a major determinant of anastomotic healing and burst strength. Conversely, empiric antibiotic treatment in other animal models prevents anastomotic leak when pathogenic bacteria or pathologic conditions are present [21]. This latter observation suggests that tissue injury alone, and the physiologic stress it incurs, may significantly influence the microbiome.

Our results indicate that intestinal resection and anastomosis induce a change in the microbiota associated with intestinal tissues, which is not observed in the luminal contents. Our findings also predict that tissue-associated microbial communities express an adhesive phenotype, which may explain, in part, their tropism toward injured tissues. Previous work from our laboratory has indicated that soluble products released during surgical injury can act as both chemoattractants and ‘cues’ that induce a phenotype shift among pathogenic bacteria such as *P. aeruginosa*[22,23]. In the present study, anastomotic tissues may select for microbes that express enhanced virulence (i.e., *E. faecalis*) and thus may have the potential to complicate anastomotic healing. A critical loss of the cytoprotective microbiota (i.e., Firmicutes and Bacteroidetes) on anastomotic tissues may further facilitate colonization and invasion by disease-associated microbiota, again with the potential to complicate healing. The precise explanation by which the process of surgery in the present study shifted the intestinal microbiota remains to be clarified. The surgery performed in the present study included a large abdominal incision, colon resection, anastomosis, and wound closure, all of which has been shown to result in a catabolic stress and immune activation as a compensatory mechanism to deal with the process of healing the wounds [24,25]. There likely exist multiple mechanisms that drive the compositional and functional changes in the microbiota including redistribution of nutrients away from the gut to healing tissues, immune activation, and changes in luminal and mucosal oxygen status [26]. It is also possible that the microbiota ‘sense’ a dramatic change in host health status and undergo compositional and functional changes as a mechanisms of adaptation until recovery to homeostasis is complete. Further work is needed to determine precisely which of the factors predominate in this model.

It is noteworthy that most of the current antibiotic regimens used as prophylaxis agents for intestinal surgery are based on culture-based analyses of expelled stool. The limitation of this approach is that antibiotics are not based on a tissue-specific microbiome. As such, we do not know which tissue microbiota are associated with healing versus non-healing. This may be critically important to prevent complications such as anastomotic leaks, which still persist today at unacceptable rates even under the most expert of care [27]. While antibiotic use has been demonstrated to be highly effective in preventing anastomotic leak, the type, duration, and microbial targets of currently recommended antibiotics have remained empirical and invalidated. Data from the present study would suggest that direct genomic and functional analyses of the tissue-associated microbiota at anastomotic sites may provide a more directed approach to antibiotic use in anastomotic surgery by understanding their effect on the microbial community structure, membership, and function.

The observed 500-fold increase in *Enterococcus* in postoperative anastomotic tissues is interesting. Although enterococci are commensal inhabitants of the mammal gut, they have been observed to be regularly associated with human infections [28]. In terms of intestinal anastomoses, it has been reported that *E. faecalis* is highly prevalent at leaking anastomotic tissues, although its role as a causative agent in leakage remains unknown [29,30]. *Enterococcus* has a high adherence affinity to extracellular cellular matrix proteins such as fibronectin, laminin, and various different types of collagens [31-33] including collagen IV [34]. What role, if any, *Enterococcus* plays in the etiopathogenesis of anastomotic leak will require further study. However, the fact that its abundance is increased to such a high degree raises the possibility that it may complicate healing given that it is known to confer a high degree of pathogenicity when it predominates. In fact, PICRUSt analysis [6] in the current work predicted the capacity of anastomotic tissues-associated microflora to possess coccolysin known as a gelatinase GelE of *E. faecalis* that is able to degrade collagen. Similarly, *Escherichia* genus also displayed a dramatic increase in relative abundance (i.e., 200-fold) at anastomotic tissues which is another commensal capable of confer a pathogenic phenotype [35]. Further work will be necessary to independently assess the virulence potential of strains isolated from anastomotic tissues versus luminal contents in both cases.

Given that the normal microbiota provide pathogen colonization resistance [36-38], there may be value in understanding how the microbiota assemble from the lumen to anastomotic tissues over the course of anastomotic healing. For example, our results demonstrated that there is a postoperative decrease in *Ruminococcaceae*, a family of Firmicutes that belong to obligate anaerobes. It has been recently shown that the families *Ruminococcaceae* and *Lachnospiraceae* (Clostridium cluster XIV group) colonize mucosal folds in the proximal murine colon [5]. Taken together, our data indicate that a significant membership of the normal microbiota may be lost at anastomotic tissues, the consequence of which is unknown. The family *Lactobacillaceae* was also reduced at anastomotic tissues and its known cytoprotective action [39-41] to accelerate healing may play a role in leak when they are critically decreased and additional physiologic stress occurs.

Although taxonomic profiling by the 16S rRNA analysis can be very informative, it does not provide any functional context to the bacterial communities. Metagenomics and metatranscriptomics would provide greater detail on their potential and actual functions yet are still technically challenging in terms of analysis. However, using PICRUSt [6], we observed predicted global changes in metabolic and virulence functions of bacterial communities among microbiota associated with anastomotic tissues. These findings provide a path forward in which we can apply this technique to models of anastomotic leak and determine its predictive capacity and its ability to inform a microbial mechanism for anastomotic leakage.

## Conclusion

An anastomotic injury appears to significantly alter the tissue-associated microbiota without affecting the luminal microbiota. A more detailed analysis of the tissues associated microbiota over the full course of anastomotic healing could lead to novel strategies for a leak prevention that may include allowing for a balance of microbial community structure, membership, and function.

## Methods

### Rat model of anastomosis

The Institute for Animal Care and Use Committee at the University of Chicago approved all animal experiments. Adult, male Wistar rats 250–300 g (Charles River Laboratory, Chicago, IL, USA) were used for all experiments and were allowed unrestricted access to rat chow and tap water. Prior to surgery, rats were sedated with 40–80 mg/kg ketamine, 5–10 mg/kg xylazine via an intraperitoneal injection. Using aseptic technique, rats were subjected to an abdominal incision (laparotomy) and a 0.5-cm distal colon resection followed by an end-to-end rectosigmoid reconnection (anastomosis) using 12 interrupted 6–0 non-absorbable sutures. Colon luminal content was collected for DNA isolation. The resected piece of colon was washed with saline and then swabbed for tissue associated microbiota. In all cases, anastomotic integrity was confirmed immediately after construction by injecting saline into the rectum and observing that the anastomotic was water tight. On postoperative day 6 all rats were sacrificed and the luminal contents adjacent to anastomosis were collected. For tissue-associated microbiota, anastomotic tissues were removed, and mechanically homogenized, and then DNA was extracted.

### 16S rRNA analysis of bacterial composition

The following are the four groups of samples in our studies: (1) POD0 tissue for which the resected piece of colon was washed with saline and then swabbed for tissue associated microbiota; (2) POD0 luminal bacteria representing luminal content from a resected piece of colon; (3) POD6 tissue for which tissues at the site of anastomosis were collected, washed with saline, and then swabbed; (4) POD6 luminal bacteria representing luminal content from collected piece of anastomotic site of the colon. Twenty rats were included in the experiment. Samples with sequences containing low confidence base calls (phred < 20) were eliminated. Samples were analyzed by 16S rRNA V4 iTAG amplicon sequencing analysis. Following joining of paired 2 × 150 bp MiSeq read sequences (Illumina, San Diego, CA, USA) containing low confidence base calls (phred < 20) were dropped, and the remaining sequences clustered into 97% similarity operational taxonomic units (OTUs) using uclust [42,43]. After dropping clusters composed of a single sequence, OTUs were assigned taxonomic annotations by aligning representative members of each cluster to the greengenes 12_04 database using the RDP classifier [44,45]. Sequencing depth among samples was normalized by rarefaction to a depth of 3,000 sequences per sample. Along with clustering, taxonomic assignment, and rarefaction, PCoA plots were derived from weighted unifrac distance matrices using the QIIME v1.6.0 software suite [46]. Significant differences in the abundance of taxonomic groups between treatments were calculated with Student’s *t* test followed by the Benjamini-Hochberg correction for multiple comparisons using an alpha of 0.05 [47]. Taxa tree was generated using GraPhlAn.

### Functional analysis of bacterial composition

We predicted the functional composition and abundance of the microbial community using PICRUSt on the OTUs derived from the 16S rRNA analysis [6]. Differential functional abundance between postoperative day 0 and day 6 for stool contents and anastomosis tissue was performed using the Wilcoxon rank sum statistical test, and *p* values were Bonferroni corrected for multiple testing. The metagenomic predictions produced by PICRUSt were further analyzed by HUMAnN [48] to provide KEGG metabolic pathway coverage, and LEfSe [49] was used for comparative analysis of the differentially enriched KEGG metabolic pathways between postoperative day 0 and day 6 for stool contents and anastomosis tissue samples. OTU contribution for enriched metabolic pathways was calculated by summing the abundance contribution of each OTU for each KEGG orthology comprising a metabolic pathway, and then normalizing by the number of samples. As there are hundreds of OTUs, a threshold was used to select just the top contributing OTUs for inclusion in Figure 5. The values were scaled to a 0–1 range for each individual metabolic pathway, with 1 indicating the highest contributing OTU and 0 indicating no contribution by that OTU.

## Abbreviations

*POD*: postoperative day; *KEGG*: Kyoto Encyclopedia of Genes and Genomes (a database resource for understanding high-level functions and utilities of the biological system); *PICRUSt*: Phylogenetic Investigation of Communities by Reconstruction of Unobserved States (a bioinformatics software package designed to predict metagenome functional content from marker gene (e.g., 16S rRNA) surveys and full genomes); *OTUs*: operational taxonomic units.

## Competing interests

The authors declare that they have no competing interests.

## Authors’ contributions

BDS has made substantial contributions to conception and design and acquisition of data. DPS, SC, and JAG have made substantial contributions to data analysis and interpretation of data. OZ and JCA have made substantial contributions to conception, design, interpretation of data, and writing the manuscript. All authors have been involved in drafting the manuscript. All authors read and approved the final manuscript.

## Authors’ information

Jack A Gilbert, Olga Zaborina, and John C Alverdy are senior co-authors.
